# Bile Leakage After Hepatic Resection for Hepatocellular Carcinoma: Does It Impact the Short- and Long-term Outcomes?

**DOI:** 10.1007/s11605-022-05433-7

**Published:** 2022-08-24

**Authors:** Ahmed Shehta, Ahmed Farouk, Rami Said, Ayman El Nakeeb, Ahmed Aboelenin, Mohamed Elshobary, Amgad Fouad, Ahmed Nabieh Elghawalby

**Affiliations:** Gastrointestinal Surgery Center, Department of Surgery, Faculty of Medicine, Mansoura University, Gehan Street, Mansoura, 35516 Egypt

**Keywords:** Hepatocellular carcinoma, Hepatic resection, Bile leakage, Survival

## Abstract

**Background:**

Bile leakage (BL) is one of the commonest morbidities after hepatic resection for hepatocellular carcinoma (HCC). The current study was conducted to evaluate the incidence and different predictive factors for BL after hepatic resection for HCC, and to evaluate of the impact of BL on the long-term survival outcomes.

**Methods:**

We reviewed the patients’ data who underwent hepatic resection for HCC during the period between June 2010 and June 2019.

**Results:**

A total of 293 patients were included in the study. BL occurred in 17 patients (5.8%). More Child–Pugh class B patients were found in BL group. There were no significant differences between the two groups except for tumor site, macroscopic portal vein invasion, extent of liver resection, Pringle maneuver use, intraoperative blood loss, and transfusions. Longer hospital stay, higher grades of post-hepatectomy liver failure, and abdominal collections were noted in BL group. After median follow-up duration of 17 months (4–110 months), there were no significant differences between BL and non-BL group regarding overall survival (log-rank, *p* = 0.746) and disease-free survival (log-rank, *p* = 0.348). In multivariate analysis, Child–Pugh class, macroscopic portal vein invasion, liver resection extent (minor/major), and Pringle’s maneuver use were the only significant predictors of BL.

**Conclusion:**

BL did not significantly impair the long-term outcomes after hepatic resection for HCC. Child–Pugh class, macroscopic portal vein invasion, liver resection extent (minor/major), and Pringle’s maneuver use were the main risk factors of BL in the current study.

## Introduction

Hepatic resection plays an essential role in the curative treatment of hepatocellular carcinoma (HCC) patients. With improvement of the patients’ selection criteria, refinement of surgical techniques, and advancement of perioperative care, the outcomes of hepatic resection have markedly improved in the recent years .^1,2^ The most important complications after hepatic resection for HCC include postoperative hemorrhage, post-hepatectomy liver failure, bile leakage (BL), and intra-abdominal infections.^3^

In spite of the overall decrease in the incidence of perioperative morbidities after hepatic resection, BL remains one of the most common morbidities after hepatic resection for HCC. The incidence of BL after hepatic resection remains controversial between the different studies, ranging between 2.6 and 12%.^3–5^ This attributed to the differences in the underlying hepatic parenchymal background, liver resection extent, techniques in hepatic parenchymal transection, and different modalities used for biliostasis on transection plane like hemostatic agents and fibrin glue.

BL is one of the most feared morbidities after hepatic resection for HCC. BL is associated with increased the risk for intra-abdominal collections, sepsis, and liver decompensation. Also, it may predispose to early postoperative mortality.^2^ On the other hand, the impact of biliary leakage on the long-term outcomes of hepatic resection for HCC is not well elucidated.

The current study was conducted to evaluate the incidence and different predictive factors for the development of BL after hepatic resection for HCC, defined according to the International Study Group of Liver Surgery (ISGLS), in area where hepatitis C virus (genotype 4) is the main predisposing factor for HCC development ^6^; and, also, to evaluate of the impact of BL on the survival outcomes after hepatic resection for HCC.

## Materials and Methods

### Study Design

We retrospectively reviewed the data of patients who underwent primary liver resection for pathologically confirmed HCC at Gastro-Intestinal Surgery Center, Mansoura University, Egypt, during the period between June 2010 and June 2019. Patient data were retrieved from a prospectively maintained database for all patients undergoing liver resection. An informed consent was obtained from each patient prior to surgical intervention. The study was approved by the Institutional Review Board and Local Ethical Committee at the Faculty of Medicine, Mansoura University, Egypt (Code Number: R.20.06.875).

### Preoperative Evaluation

Preoperative workup included detailed laboratory and radiological evaluation, as previously shown.^7,8^ Selection of appropriate treatment strategy was discussed at multidisciplinary meetings. Generally, liver resection was applied for patients with preserved liver functions, without signs of severe portal hypertension, without evidence of extrahepatic metastasis, and with American Society of Anesthesiologists (ASA) grade < III.^9^

### Surgical Procedure

The surgical procedure had been described previously.^7,8,10^ Generally, parenchymal sparing liver resection was preferred. Major liver resections were performed for patients with large tumors or tumors close to major hepatic vasculature if the future remnant liver is adequate (more than 40% of the total liver volume). Volumetric assessment was performed for selected patients requiring major liver resection with marginal liver functions. Otherwise, non-anatomical liver resections were more preferred.

Parenchymatous transection was performed by combinations of clamp-crush method and ultrasonic devices. Intermittent Pringle’s maneuver was applied selectively during liver transection. Intraoperative ultrasonography was utilized in some patients to check the resection margin and exclude presence of multifocal tumors. Intraoperative cholangiography was performed in some patients to ensure biliostasis and assess the remnant biliary system integrity.

### Postoperative Care and Follow-up

After surgery, patients were transferred to the intensive care unit or to the ward for monitoring of vital signs and routinely inserted abdominal drains. All patients underwent daily laboratory evaluation. Abdominal ultrasonography was performed routinely in all patients. Oral fluids were started once intestinal sounds were restored. Abdominal drains were removed when daily output was less than 100 cc with absence of any abdominal collections. After discharge, patients were followed up in the outpatient clinic. Follow-up visit included routine laboratory and radiological evaluation.

### Study Outcomes

The primary outcome of the study is the incidence of BL, defined according to the International Study Group of Liver Surgery (ISGLS).^6^ Secondary outcomes included evaluation of the impact of BL on overall survival (OS) and disease-free survival (DFS), also to evaluate different predictive factors for the development of BL after liver transection for HCC.

### Definitions

The types of liver resection were defined according to Brisbane 2000 terminology.^11^ Liver resections were classified into minor (≤ 2 segments) or major (≥ 3 segments) according to Couinaud classification. Postoperative morbidity is defined as adverse events happening during the early postoperative period and is graded according to the Clavien-Dindo classification.^12^ Postoperative BL was defined according to the International Study Group of Liver Surgery (ISGLS).^6^ Post-hepatectomy liver failure was defined according to the ISGLS definition.^13^

Early postoperative mortality was defined as mortality occurring during the first 90 postoperative days, and was excluded from further survival analysis. OS was calculated from the day of surgery to the day of confirmed death or the last follow-up visit. DFS was calculated from the day of surgery to the day of confirmed tumor recurrence or the day of death or last follow-up.

### Statistical Analysis

Shapiro–Wilk test is used to assess the normality of continuous data. Categorical variables are expressed as number and percentage, and continuous variables are expressed as median and range. Comparison between groups is done by chi-square test for categorical variables and Mann–Whitney test for continuous variables. Survival analysis is performed by Kaplan–Meier method and comparison between groups is done by log-rank test.

Univariate and multivariate analyses are done by binary logistic regression analysis to identify the independent risk factors for BL. Significant factors determined in the univariate analysis are included in the subsequent multivariate analysis. Statistical analysis of the data is performed using IBM-SPSS software for Windows, version 24 (IBM Corp., Armonk, NY). A *p* value < 0.05 is considered significant.

## Results

During the period between June 2010 and June 2019, 293 patients underwent liver resection for HCC at Gastro-Intestinal Surgery Center, Mansoura University, Egypt. Patients were divided into two groups according to the occurrence of BL, non-bile leakage (non-BL) group (276 patients 94.2%), and bile leakage (BL) group (17 patients 5.8%).

### Demographic Data

Demographic data of the study patients are shown in Table 1. There were no significant differences between the two groups except for Child–Pugh score. More Child–Pugh class A patients were found in non-BL group while more Child–Pugh class B patients were found in BL group. Hepatitis C virus infection was the most common underlying cause for HCC among the study patients.

**Table 1:** Demographic data of the study patients (*TACE*, transarterial chemoembolization; *RFA*, radiofrequency ablation)

Variables	All cases (*N* = 293)	Non-BL group (*N* = 276)	BL group (*N* = 17)	*P* value
Age (years)	60 (18–78)	60 (18–78)	59 (45–68)	0.947
Gender Male Female	237 (80.9%) 56 (19.1%)	222 (80.4%) 54 (19.6%)	15 (88.2%) 2 (11.8%)	0.542
Body mass index (kg/m^2^)	28.7 (17.3–42.7)	28.7 (17.3–42.7)	27.9 (20.8–38.5)	0.942
Previous abdominal operations	92 (31.4%)	89 (32.2%)	3 (17.6%)	0.285
Previous TACE	18 (6.1%)	18 (6.5%)	0	0.61
Previous RFA	4 (1.4%)	4 (1.4%)	0	1
Complaint Accidental Pain Mass	141 (48.1%) 150 (51.2%) 2 (0.7%)	136 (49.3%) 138 (50%) 2 (0.7%)	5 (29.4%) 12 (70.6%) 0	0.251
Previous antiviral therapy	29 (9.9%)	28 (10.1%)	1 (5.9%)	1
Albumin (g/dL)	3.9 (2.1–5.3)	3.9 (2.1–5.3)	3.8 (2.2–4.8)	0.85
Bilirubin (mg/dL)	0.7 (0.3–11.2)	0.7 (0.3–2.2)	0.7 (0.5–11.2)	0.936
Alanine aminotransferase (IU/L)	41 (20–280)	41 (20–280)	35 (20–127)	0.965
Aspartate aminotransferase (IU/L)	50 (20–240)	50 (20–240)	47 (20–236)	0.942
International normalized ratio	1 (1–1.8)	1 (1–1.8)	1 (1–1.2)	0.084
Platelets (× 10^3^/mL)	145 (34–433)	143.5 (34–433)	159 (72–294)	0.284
Creatinine (mg/dL)	0.8 (0.5–2.5)	0.8 (0.5–2.5)	0.8 (0.6–1.1)	0.906
Alpha feto-protein (ng/ml)	30.1 (3.4–2000)	29 (7–2000)	195 (3.4–2000)	0.617
Child–Pugh grade A B	286 (97.6%) 7 (2.4%)	272 (98.6%) 4 (1.4%)	14 (82.4%) 3 (17.6%)	0.005
Model for end stage liver disease (MELD score)	7 (6–16)	7 (6–16)	7 (6–16)	0.718
Hepatitis C virus	270 (92.2%)	254 (92%)	16 (94.1%)	1
Hepatitis B virus	3 (1%)	2 (0.7%)	1 (5.9%)	0.165

### Radiological and Endoscopic Data

Radiological and endoscopic data of the study patients are summarized in Table 2. There were no significant differences between the groups regarding preoperative radiological and endoscopic data apart from presence of macroscopic portal vein invasion. Macroscopic portal vein invasion was more observed in BL group (5 patients 29.4%) compared to non-BL groups (31 patients 11.2%) (*p* = 0.043).

**Table 2:** Radiological and endoscopic data of the study patients

Variables	All cases (*N* = 293)	Non-BL group (*N* = 276)	BL group (*N* = 17)	*P* value
Liver status Cirrhosis Normal	278 (94.9%) 15 (5.1%)	262 (94.9%) 14 (5.1%)	16 (94.1%) 1 (5.9%)	0.601
Spleen Normal Mild splenomegaly Moderate splenomegaly Marked splenomegaly Absent	122 (41.6%) 123 (42%) 38 (13%) 3 (1%) 7 (2.4%)	112 (40.6%) 118 (42.8%) 36 (13%) 3 (1.1%) 7 (2.5%)	10 (58.8%) 5 (29.4%) 2 (11.8%) 0 0	0.628
Number Single Multiple	266 (90.8%) 27 (9.2%)	249 (90.2%) 27 (9.8%)	17 (100%) 0	0.382
Site Right hemi-liver Left hemi-liver Left lateral section Segment IV Right anterior section Right posterior section Central Caudate lobe Segment II Segment III Segment V Segment VI Segment VII Segment VIII Multi-site	27 (9.2%) 14 (4.8%) 38 (13%) 12 (4.1%) 3 (1%) 14 (4.8%) 4 (1.4%) 6 (2%) 22 (7.5%) 34 (11.6%) 13 (4.4%) 45 (15.4%) 26 (8.9%) 16 (5.5%) 19 (6.5%)	23 (8.3%) 13 (4.7%) 36 (13%) 12 (4.3%) 3 (1.1%) 12 (4.3%) 4 (1.4%) 6 (2.2%) 22 (8%) 33 (12%) 11 (4%) 45 (16.3%) 24 (8.7%) 13 (4.7%) 19 (6.9%)	4 (23.5%) 1 (5.9%) 2 (11.8%) 0 0 2 (11.8%) 0 0 0 1 (5.9%) 2 (11.8%) 0 2 (11.8%) 3 (17.6%) 0	0.114
Size (cm)	6 (1.7–20)	6 (1.7–20)	6.3 (3–12)	0.498
Macroscopic portal vein invasion	36 (12.3%)	31 (11.2%)	5 (29.4%)	0.043
Porta hepatis lymph nodes	44 (15%)	41 (14.9%)	3 (17.6%)	0.727
Upper GIT endoscopy	284 (96.9%)	267 (96.7%)	17 (100%)	1
Endoscopy findings Esophageal veins Gastric compression	50 (17.1%) 1 (0.3%)	50 (18.1%) 1 (0.4%)	0 0	0.144

### Operative Data

Operative data of the study patients are summarized in Table 3. There were significant differences between the two groups regarding tumor site, macroscopic portal vein invasion, extent of liver resection, type of liver resection, Pringle maneuver use, intraoperative blood loss, and blood transfusions.

**Table 3:** Operative data of the study patients (*HCC*, hepatocellular carcinoma; *LGL*, left gastric ligation; *IO RFA*, intraoperative radiofrequency ablation)

Variables	All cases (*N* = 293)	Non-BL group (*N* = 276)	BL group (*N* = 17)	*P* value
Liver status Cirrhosis Normal	274 (93.5%) 19 (6.5%)	259 (93.8%) 17 (6.2%)	15 (88.2%) 2 (11.8%)	0.303
Site Right hemi-liver Left hemi-liver Caudate lobe Bilobar	151 (51.5%) 130 (44.4%) 6 (2%) 6 (2%)	138 (50%) 126 (45.7%) 6 (2.2%) 6 (2.2%)	13 (76.5%) 4 (23.5%) 0 0	0.197
Lesion site details Right hemi-liver Left hemi-liver Left lateral section Segment IV Right anterior section Right posterior section Central Caudate lobe Segment II Segment III Segment V Segment VI Segment VII Segment VIII Multi-site	24 (8.2%) 10 (3.4%) 54 (18.4%) 13 (4.4%) 2 (0.7%) 12 (4.1%) 10 (3.4%) 6 (2%) 11 (3.8%) 32 (10.9%) 13 (4.4%) 42 (14.3%) 30 (10.2%) 15 (5.1%) 19 (6.4%)	22 (8%) 8 (2.9%) 52 (18.8%) 13 (4.7%) 1 (0.4%) 9 (3.3%) 10 (3.6%) 6 (2.2%) 11 (4%) 32 (11.6%) 11 (4%) 42 (15.2%) 27 (9.8%) 14 (5.1%) 18 (6.6%)	2 (11.8%) 2 (11.8%) 2 (11.8%) 0 1 (5.9%) 3 (17.6%) 0 0 0 0 2 (11.8%) 0 3 (17.6%) 1 (5.9%) 1 (5.9%)	0.008
Number Single Multiple	271 (92.5%) 22 (7.5%)	256 (92.8%) 20 (7.2%)	15 (88.2%) 2 (11.8%)	0.371
Size (cm)	6 (2–20)	6 (2–20)	6 (3–10)	0.523
Vascular invasion	39 (13.3%)	34 (12.3%)	5 (29.4%)	0.049
Biliary invasion	1 (0.3%)	0	1 (5.9%)	0.058
Nearby organ invasion	19 (6.5%)	18 (6.5%)	1 (5.9%)	1
Lymph nodes	25 (8.5%)	22 (8%)	3 (17.6%)	0.168
Lymph nodes site Porta-hepatis Supra-duodenal	22 (7.5%) 2 (0.7%)	19 (6.9%) 2 (0.7%)	3 (17.6%) 0	1
Intraoperative biopsies	16 (5.5%)	14 (5.1%)	2 (11.8%)	0.235
Biopsy site Suspicious liver nodule Safety margin Lymph nodes	3 (1%) 5 (1.7%) 8 (.7%)	3 (1.1%) 5 (1.8%) 6 (2.2%)	0 0 2 (11.8%)	0.515
Biopsy result HCC High-grade tumor Negative	5 (1.7%) 1 (0.3%) 10 (3.4%)	5 (1.8%) 1 (0.4%) 8 (.9%)	0 0 2 (11.8%)	0.504
Surgery approach Open Laparoscopic Failed laparoscopic	287 (98%) 4 (1.4%) 2 (0.7%)	270 (97.8%) 4 (1.4%) 2 (0.7%)	17 (100%) 0 0	0.828
Liver resection extent Minor Major	224 (76.5%) 69 (23.5%)	219 (79.3%) 57 (20.7%)	5 (29.4%) 12 (70.6%)	0.001
Liver resection type Tumorectomy Segmentectomy Left lateral sectionectomy Right anterior sectionectomy Right posterior sectionectomy Left hepatectomy Extended left hepatectomy Right hepatectomy Extended right hepatectomy Central hepatectomy Caudate lobectomy Multiple resections	139 (47.4%) 6 (2%) 66 (22.5%) 1 (0.3%) 1 (0.3%) 13 (4.4%) 1 (0.3%) 50 (17.1%) 5 (1.7%) 1 (0.3%) 6 (2%) 4 (1.4%)	137 (49.6%) 5 (1.8%) 64 (23.2%) 1 (0.4%) 1 (0.4%) 11 (4%) 1 (0.4%) 41 (14.9%) 4 (1.4%) 1 (0.4%) 6 (2.2%) 4 (1.4%)	2 (11.8%) 1 (5.9%) 2 (11.8%) 0 0 2 (11.8%) 0 9 (52.9%) 1 (5.9%) 0 0 0	0.008
Associated portal thrombectomy	6 (1.4%)	5 (1.8%)	1 (5.9%)	0.304
Associated extrahepatic biliary resection	1 (0.3%)	0	1 (5.9%)	0.058
Pringle procedure use	44 (15%)	37 (13.4%)	7 (41.2%)	0.007
Pringle indication Elective Emergency	27 (9.2%) 17 (5.8%)	23 (8.3%) 14 (5.1%)	4 (23.5%) 3 (17.6%)	1
Pringle duration (minutes)	17.5 (10–90)	20 (10–90)	15 (10–30)	1
Operation time (hours)	3 (1.2–7)	3 (1.2–6)	4 (3–7)	0.001
Blood loss (ml)	600 (50–6000)	600 (50–6000)	1300 (200–4000)	0.012
Blood transfusion	144 (49.1%)	132 (47.8%)	12 (70.6%)	0.082
Associated operation	82 (28%)	79 (28.6%)	3 (17.6%)	0.414

### Postoperative Data

Postoperative data of the study patients are summarized in Table 4. Longer hospital stay was noted in BL group (15 vs 5 days, *p* = 0.001). Higher grades of post-hepatectomy liver failure (grade B and C) and postoperative abdominal collections were noted in BL group.

**Table 4:** Postoperative data of the study patients (*ICU*, intensive care unit; *PHLF*, post-hepatectomy liver failure; *US*, ultrasound; *ERCP*, endoscopic retrograde cholangio-pancreatography)

Variables	All cases (*N* = 293)	Non-BL group (*N* = 276)	BL group (*N* = 17)	*P* value
ICU duration (days)	1 (1–22)	1 (1–22)	1 (1–2)	0.972
Hospital stay (days)	5 (2–66)	5 (2–31)	15 (7–66)	0.001
Morbidity	153 (52.2%)	136 (49.3%)	17 (100%)	0.001
Clavien-Dindo grade I II III-a III-b IV-a V	64 (21.8%) 47 (16%) 12 (4.1%) 10 (3.4%) 2 (0.7%) 18 (6.1%)	61 (22.1%) 45 (16.3%) 8 (2.9%) 5 (1.8%) 0 17 (6.2%)	3 (17.6%) 2 (11.8%) 4 (23.5%) 5 (29.4%) 2 (11.8%) 1 (5.9%)	0.001
Morbidity type General Surgical Liver-related Mixed	4 (1.4%) 7 (2.4%) 108 (36.9%) 34 (11.6%)	4 (1.4%) 6 (2.2%) 100 (36.2%) 26 (9.4%)	0 1 (5.9%) 8 (47.1%) 8 (47.1%)	0.061
PHLF	138 (47.1%)	127 (46%)	11 (64.7%)	0.143
PHLF grade A B C	77 (26.3%) 41 (14%) 20 (6.8%)	75 (27.2%) 35 (12.7%) 17 (6.2%)	2 (11.8%) 6 (35.3%) 3 (17.6%)	0.032
Bile leakage	17 (5.8%)	0	17 (100%)	–-
Bile leakage treatment Conservative US-guided tube ERCP Operative	6 (2%) 3 (1%) 7 (2.4%) 1 (0.3%)	0	6 (35.3%) 3 (17.6%) 7 (41.2%) 1 (5.9%)	–-
Collection	14 (4.8%)	11 (4%)	3 (17.6%)	0.04
Collection treatment Conservative US-guided tube Operative	6 (2%) 7 (2.4%) 1 (0.3%)	6 (2.2%) 5 (1.8%) 0	0 2 (11.8%) 1 (5.9%)	0.063
Internal hemorrhage Managed surgically	6 (2%)	6 (2.2%)	0	1
Wound infection All bed side management	9 (3.1%)	7 (2.5%)	2 (11.8%)	0.09
Liver abscess All US-guided drainage	3 (1%)	2 (0.7%)	1 (5.9%)	0.165
Vascular complications All PVT	5 (1.7%)	5 (1.8%)	0	1
Respiratory complications Pleural effusion Pneumonia	17 (5.8%) 16 (5.5%) 1 (0.3%)	14 (5.1%) 13 (4.7%) 1 (0.4%)	3 (17.6%) 3 (17.6%) 0	0.066
Respiratory treatment Conservative US-guided drainage	13 (4.4%) 4 (1.4%)	11 (4%) 3 (1.1%)	2 (11.8%) 1 (5.9%)	1
Cardiac dysrhythmia	1 (0.3%)	1 (0.4%)	0	1
Renal complications All hepato-renal syndrome	4 (1.4%)	4 (1.4%)	0	1
Cerebral stroke	1 (0.3%)	1 (0.4%)	0	1
Ileus	1 (0.3%)	1 (0.4%)	0	1
Bleeding varices Endoscopic management	1 (0.3%)	1 (0.4%)	0	1
Early mortality	20 (6.8%)	19 (6.9%)	1 (5.9%)	1
Early mortality cause Liver failure	20 (6.8%)	19 (6.9%)	1 (5.9%)	1

### Pathological Outcomes

Pathological data of the study patients are summarized in Table 5. There were no significant differences between the two groups regarding different pathological data.

**Table 5:** Pathological data of the study patients

Variables	All cases (*N* = 293)	Non-BL group (*N* = 276)	BL group (*N* = 17)	*P* value
Size (cm)	6 (1.5–20)	6 (1.5–20)	5.5 (2.5–16)	0.96
Number Single Multiple	254 (86.7%) 39 (13.3%)	238 (86.2%) 38 (13.8%)	16 (94.1%) 1 (5.9%)	0.71
Resection margin R0 R1	259 (88.4%) 34 (11.6%)	244 (88.4%) 32 (11.6%)	15 (88.2%) 2 (11.8%)	1
Capsular invasion	109 (37.2%)	103 (37.3%)	6 (35.3%)	1
Microvascular invasion	140 (47.8%)	131 (47.5%)	9 (52.9%)	0.804
Perineural invasion	119 (40.6%)	111 (40.2%)	8 (47.1%)	0.617
Tumor grade I II III IV No viable tumor	55 (18.8%) 170 (58%) 59 (20.1%) 8 (2.7%) 1 (0.3%)	51 (18.5%) 163 (59.1%) 55 (19.9%) 6 (2.2%) 1 (0.4%)	4 (23.5%) 7 (41.2%) 4 (23.5%) 2 (11.8%) 0	0.155
Tumor stage T1 T2 T3 T4 Tx	75 (25.6%) 174 (59.4%) 39 (13.3%) 4 (1.4%) 1 (0.3%)	73 (26.4%) 163 (59.1%) 36 (13%) 3 (1.1%) 1 (0.4%)	2 (11.8%) 11 (64.7%) 3 (17.6%) 1 (5.9%) 0	0.349
Liver background Cirrhosis Normal	276 (94.2%) 17 (5.8%)	259 (93.8%) 17 (6.2%)	17 (100%) 0	0.609

### Survival Outcomes

#### Overall Survival

**Fig. 1:**
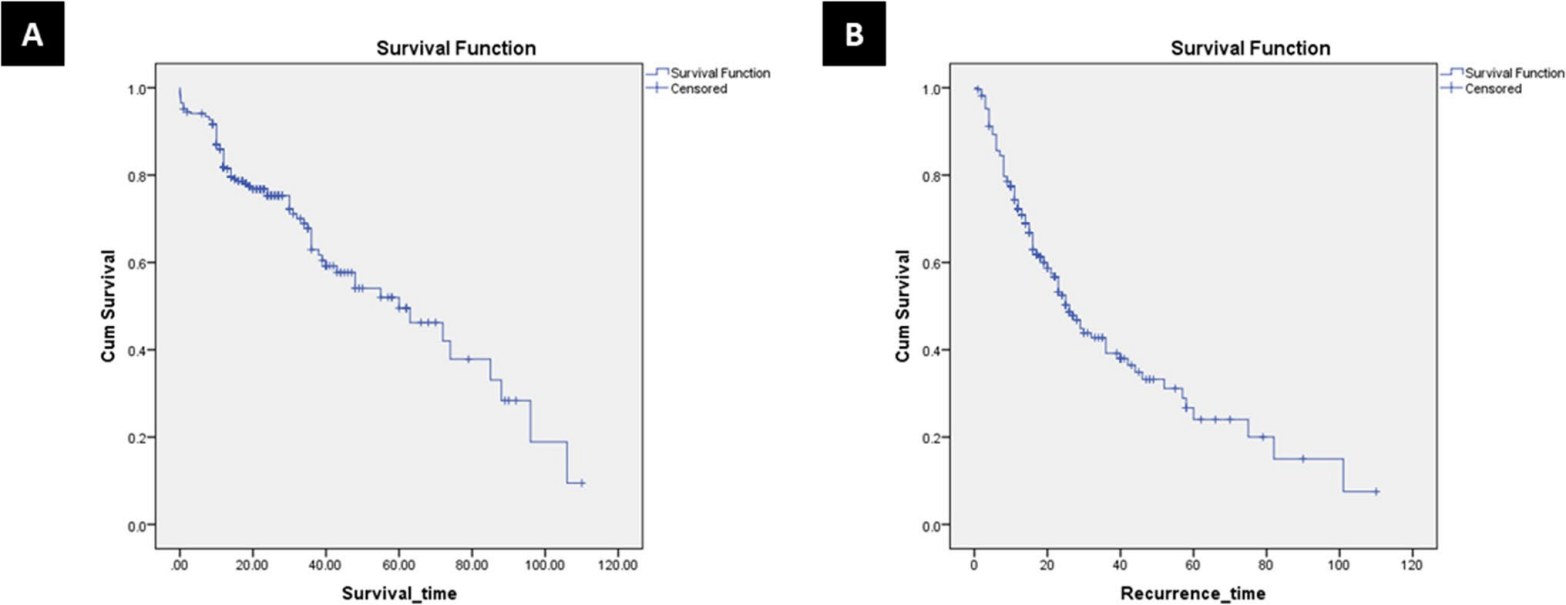
**A** Kaplan–Meier overall survival curve of all study cases. **B** Kaplan–Meier disease-free survival curve of all study cases

**Fig. 2:**
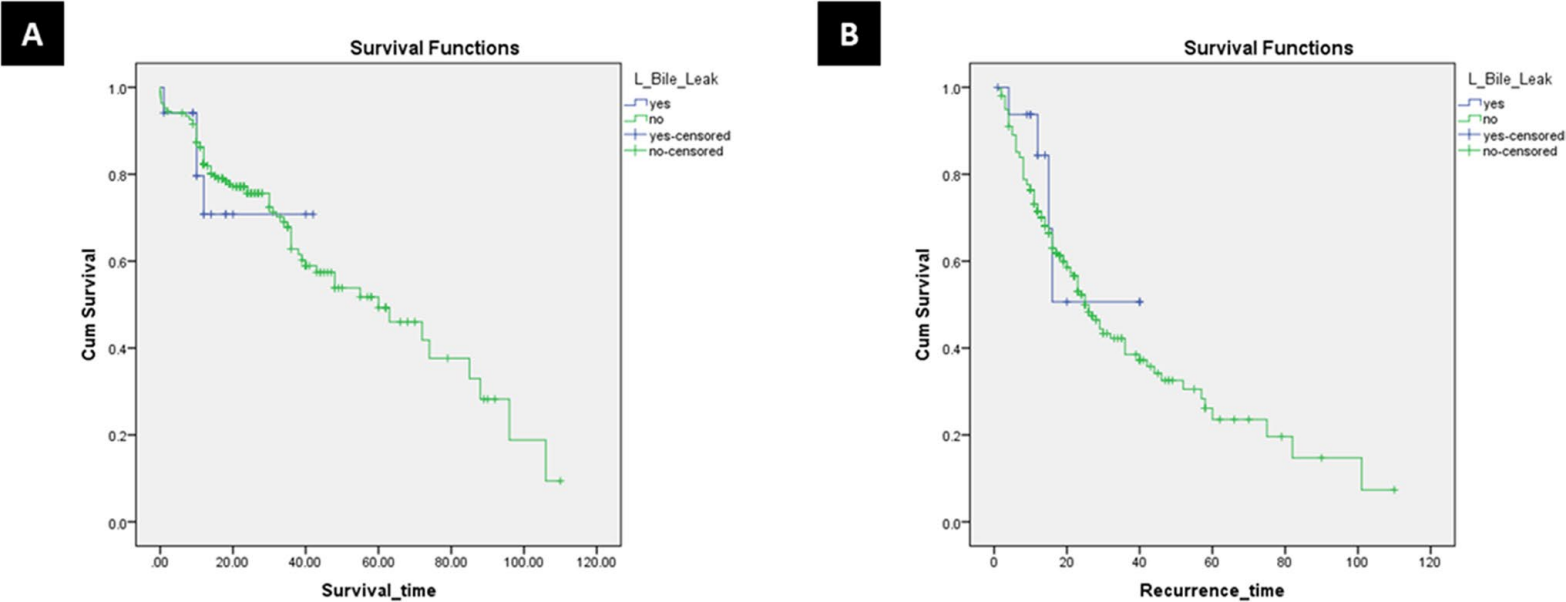
**A** Kaplan–Meier overall survival curve of both groups (log-rank; chi square 0.105–df: 1–*p* = 0.746). **B** Kaplan–Meier disease-free survival curve of both groups (log-rank; chi square 0.881–df: 1–*p* = 0.348)

The median follow-up duration was 17 months (4–110 months). Mortality occurred in 89 patients (30.4%). The 1-, 3-, and 5-year OS rates for all study patients were 85.9%, 68.6%, and 49.5%, respectively (Fig. 1A). The 1-, 3-, and 5-year OS rates for the non-BL group were 86.2%, 67.8%, and 49.3%, respectively. The 1-, 3-, and 5-year OS rates for BL group were 79.6%, 70.8%, and 70.8%, respectively (log-rank, *p* = 0.746) (Fig. 2A).

#### Disease-Free Survival

Recurrence occurred in 133 patients (45.4%). There were no significant differences between the groups regarding recurrence time, and recurrence management as shown in Table 6. More extrahepatic recurrences occurred in BL group (*p* = 0.017). The 1-, 3-, and 5-year DFS rates for all study patients were 74.3%, 42.8%, and 26.7%, respectively (Fig. 1B). The 1-, 3-, and 5-year DFS rates for the non-BL group were 73.2%, 42.2%, and 26.2%, respectively. The 1-, 3-, and 5-year DFS rates for BL group were 84.4%, 50.6%, and 50.6%, respectively (log-rank, *p* = 0.348) (Fig. 2B).

**Table 6:** Recurrence and survival data of the study patients

Variables	All cases (*N* = 293)	Non-BL group (*N* = 276)	BL group (*N* = 17)	*P* value
Mortality	89 (30.4%)	85 (30.8%)	4 (23.5%)	0.598
Survival time (month)	17 (4–110)	17 (4–110)	12 (4–42)	0.167
Overall survival 1 year 3 years 5 years	85.9% 68.9% 49.5%	86.2% 67.8% 49.3%	79.6% 70.8% 70.8%	Log-rank 0.746
Recurrence	133 (45.4%)	129 (46.7%)	4 (23.5%)	0.044
Recurrence time (month)	14 (1–110)	14 (1–110)	12 (1–40)	0.204
Disease-free survival 1 year 3 years 5 years	74.3% 42.8% 26.7%	73.2% 42.2% 26.2%	84.4% 50.6% 50.6%	Log-rank 0.348
Recurrence site Intrahepatic Extrahepatic Both	101 (34.5%) 5 (1.7%) 27 (9.2%)	100 (36.2%) 4 (1.4%) 25 (9.1%)	1 (5.9%) 1 (5.9%) 2 (11.8%)	0.017
Intrahepatic site Liver margin Same liver lobe Other liver lobe Bilobar	4 (1.4%) 30 (10.2%) 41 (14%) 53 (18.1%)	4 (1.4%) 29 (10.5%) 39 (14.1%) 53 (19.2%)	0 1 (5.9%) 2 (11.8%) 0	0.449
Intrahepatic treatment Resection TACE RFA MWA Combined therapy Systemic therapy Supportive	2 (0.7%) 37 (12.6%) 9 (3.1%) 7 (2.4%) 12 (4.1%) 1 (0.3%) 60 (20.5%)	2 (0.7%) 37 (13.4%) 9 (3.3%) 7 (2.5%) 12 (4.3%) 1 (0.4%) 57 (20.7%)	0 0 0 0 0 0 3 (17.6%)	0.968
Extrahepatic site Lung Bone Brain Peritoneum Adrenal gland Abdominal wall Lymph nodes Multi-site	12 (4.1%) 5 (1.7%) 1 (0.3%) 5 (1.7%) 1 (0.3%) 1 (0.3%) 2 (0.7%) 5 (1.7%)	10 (3.6%) 5 (1.8%) 1 (0.4%) 5 (1.8%) 1 (0.4%) 0 2 (0.7%) 2 (0.7%)	2 (11.8%) 0 0 0 0 1 (5.9%) 0 0	0.135

### Predictive Factors for Bile Leakage

Predictive factors for BL are shown in Table 7. In univariate analysis, Child–Pugh class, tumor site, macroscopic portal vein invasion, liver resection extent (minor/major), Pringle’s maneuver, and operation time were significantly correlated with BL. In multivariate analysis, Child–Pugh class, macroscopic portal vein invasion, liver resection extent (minor/major), and Pringle’s maneuver were the only significant predictors of BL.

**Table 7:** Predictive factors of biliary leakage (*TACE*, transarterial chemoembolization; *RFA*, radiofrequency ablation; *MELD*, model for end stage liver disease)

Variables	Univariate analysis		Multivariate analysis	
	HR (95% CI)	*P* value	HR (95% CI)	*P* value
Age	1.001 (0.001–0.031)	0.984		
Gender	0.601 (0.601–0.768)	0.434		
Previous TACE	18.48 (18.29–94.605)	0.998		
Previous RFA	18.43 (0.25–122.996)	0.999		
Albumin	0.067 (0.518–0.016)	0.898		
Bilirubin	0.456 (0.261–3.051)	0.081		
Alanine aminotransferase	0.001 (0.007–0.013)	0.909		
Aspartate aminotransferase	0.005 (0.006–0.723)	0.935		
International normalized ratio	6.264 (3.608–3.014)	0.083		
Platelets	0.003 (0.003–0.848)	0.357		
Creatinine	0.453 (0.991–0.209)	0.647		
Alpha feto-protein	0 (0–1.847)	0.174		
Child–Pugh grade	2.679 (0.142–0.764)	0.001	2.502 (0.762–0.906)	0.006
MELD score	0.022 (0.148–1.124)	0.88		
Hepatitis C virus	0.326 (0.096–1.054)	0.757		
Hepatitis B virus	2.147 (1.251–2.944)	0.086		
Tumor Site	1.131 (0.566–0.75)	0.046	0.174 (0.593–0.086)	0.769
Portal vein invasion	1.19 (0.296–0.565)	0.035	0.404 (0.667–0.3.67)	0.545
Biliary invasion	24.051 (4.254–45.559)	1		
Liver resection extent (minor vs major)	1.558 (0.318–0.553)	0.001	1.813 (0.295–0.788)	0.021
Pringle procedure	1.509 (0.524–0.885)	0.004	1.592 (0.625–1.045)	0.011
Operation time	0.818 (0.208–0.883)	0.001	0.301 (0.295–4.75)	0.307
Blood loss	3.168 (0.359–3.032)	0.082		
Blood transfusion	0.962 (0.546–3.11)	0.078		
Morbidity	19.123 (3.925–339.276)	0.996		
Morbidity grade	0.221 (0.114–3.774)	0.052		
Pathologic variant	1.385 (1.94–14.99)	0.999		
Tumor size	0.031 (0.07–1.947)	0.66		
Number (single/multiple)	0.938 (0.805–1.045)	0.37		
Resection margin (R0/R1)	0.017 (0.776–9.667)	0.983		
Capsular invasion	0.88 (0.523–10.706)	0.867		
Microvascular invasion	0.219 (0.501–9.961)	0.661		
Perineural invasion	0.279 (0.501–8.465)	0.578		
Tumor grade	0.303 (0.329–3.438)	0.358		
Tumor stage	0.537 (0.332–2.619)	0.106		
Liver background (cirrhosis/normal)	18.479 (19.496–39.682)	1		

## Discussion

With the recent advancements in the surgical techniques and perioperative patients’ care, the rate of perioperative mortality after hepatic resection for HCC has dramatically improved.^14^ However, the rates of perioperative morbidities remain a major concern. The most important complications after hepatic resection for HCC include postoperative hemorrhage, post-hepatectomy liver failure, BL, and intra-abdominal infections.^3^ BL after hepatic resection for HCC continue to be a common reason of major perioperative morbidity.^15,16^

Previous studies had shown variable incidence of BL following hepatic resection for various benign and malignant liver tumors ranging between 2.6 and 12%.^3–5,15–17^ The great differences in the BL incidence between the different studies are related to lack of standardized definition of BL after hepatic resection. The International Study Group of Liver Surgery (ISGLS) has proposed a new consensus definition of BL following hepato-biliary surgeries based on the postoperative course of bilirubin concentrations in the serum and the abdominal drainage fluid.^6^ The definition is simple, easy to apply, and enabled comparison of the results between the different clinical studies. Also, it enabled objective assessment of various treatment modalities.^6^ In the current study, BL occurred in 17 patients (5.8%). Some studies had shown a close relationship between BL and high postoperative mortality.^3,18,19^ This is attributed to high risk of septic complications associated with BL which may progress to liver failure and ascites. In the current study, we experienced higher grades of post-hepatectomy liver dysfunction and abdominal collections. However, they were carefully managed, and we did not experience any postoperative mortality due to BL. This is attributed to close monitoring and early intervention for cases with BL among our series. Six cases (35.3%) were managed conservatively, three cases (17.6%) required radiologic intervention, seven cases (41.2%) required endoscopic intervention, and only one case (5.9%) required surgical intervention. In the current study, we noticed high incidence of post-hepatectomy liver failure among our patients.^8^ We also noticed higher incidence of post-hepatectomy liver failure in patients with BL; however, this was not statistically significant. The high incidence of post-hepatectomy liver failure in BL group was more related to underlying liver parenchymal dysfunction and the extent of liver resection rather than BL.

Several previous studies analyzed different risk factors for BL after various types of hepatic resection. These risk factors include preoperative ablation, liver cirrhosis, mixed hepatocellular-cholangiocarcinoma, prolonged operation time, extended liver resections, resections involving segment IV, and repeated hepatic resection.^16,20,21^ In the current study, we found that preoperative Child–Pugh class, macroscopic portal vein invasion, liver resection extent (minor/major), and Pringle’s maneuver use were the only significant predictors of BL in multivariate analysis.

HCC usually develops on a background of liver cirrhosis. A previous study by Tanaka et al. reported that patients with liver cirrhosis experienced lower incidence BL but the difference was not statistically significant.^22^ Capussotti et al. found that patients with liver cirrhosis experienced a lower rate of BL during the postoperative course after hepatic resection.^3^ This is attributed to the extent of liver resection that is applied in patients with liver cirrhosis. Minor resections are usually applied in this group of patients, with a lower rate of extended and major resections. In the current study, we found that the degree of severity of liver cirrhosis as demonstrated by Child–Pugh score was a significant predictor for BL after hepatic resection for HCC. We also found higher incidence of child B cirrhosis in patients who experienced biliary leakage. Also, these groups of patients underwent more major hepatic resections compared to patients who did not experience biliary leakage. It has been reported that biliary leakage is more common after extended and major hepatic resections.^2^ In major hepatic resections, extended transection planes are usually utilized that is often extended out of the portal fissures and with an extensive dissection of the hepatic duct close to the hilar confluence.^23^

Several previous studies have stated that partial hepatic resection in which there is exposure of major Glissonian pedicles during parenchymatous transection such as central bisectionectomy, segment IV resection, and segment VIII resection were independent predictors of BL.^3,17,18,21^ In the current study, we found that extended liver resections (major liver resections) were significantly associated with the risk for the development of biliary leakage. However, we did not find any significant correlation of different types of liver resection and biliary leakage. Tumors requiring major and extended liver resections require transection planes that is often extended out of the portal fissures and with extensive dissection of the hepatic duct close to the hilar confluence to achieve tumor-free margins.

Also, we found that a prolonged operating time and pringle maneuver use were independent risk factors for BL after hepatic resection on univariate analysis and only pringle maneuver use on multivariate analysis. A prolonged operation time and pringle maneuver use are potential indicators of hepatic resections that are technically difficult or require complicated transection planes.

Finally, we analyzed the impact of BL on the long-term survival outcomes of liver resection for HCC. We hypothesized that patients who experienced biliary leakage will have worse long-term survival outcomes. After a median follow-up duration of 17 months (4–110 months), patients with biliary leakage experienced relatively lower overall and disease-free survival outcomes; however, this was not statistically significant. Few studies had evaluated the impact of BL on the long-term outcomes after hepatic resection especially for HCC. Concerns have been raised regarding the progression of different liver tumors after major biliary leakage following hepatic resections. Braunwarth et al. analyzed the impact of BL on the survival outcomes after hepatic resection of different liver tumors. They found a relevant influence of biliary leakage on OS in perihilar cholangiocarcinoma, whereas no association was seen in other liver tumors, indicating that tumor progression might be triggered by BL in cancer types arising from the bile ducts itself.^24^ Yamamoto et al. in a study evaluating the long-term impact of BL after liver resection for HCC found that post-operative BL was strongly associated with poorer OS and DFS following liver resection and may contribute to our understanding of the requirements for preventing BL and developing strategies towards achieving improved treatment outcomes of postoperative BL.^25^

The current study has several limitations including its retrospective nature; it included data from a single center, and it included limited number of patients. Also, the number of bile leakage events was relatively small. A future multicenter study including large number of cases from our locality is required to support our findings.

## Conclusion

BL is one of the commonest morbidities after hepatic resection for HCC. It is associated with prolonged hospital stay, higher grades of post-hepatectomy liver failure, and frequent abdominal collections. However, it is not associated with higher perioperative mortality when carefully managed. Child–Pugh class, macroscopic portal vein invasion, liver resection extent (minor/major), and Pringle’s maneuver use were the main risk factors of BL in this series of patients. BL did not significantly impair the long-term outcomes of this group of patients.
